# Pitaya Extracts Induce Growth Inhibition and Proapoptotic Effects on Human Cell Lines of Breast Cancer via Downregulation of Estrogen Receptor Gene Expression

**DOI:** 10.1155/2017/7865073

**Published:** 2017-07-06

**Authors:** Deborah de Almeida Bauer Guimarães, Danielle dos Santos Bonfim De Castro, Felipe Leite de Oliveira, Eduardo Matos Nogueira, Marco Antônio Mota da Silva, Anderson Junger Teodoro

**Affiliations:** ^1^Nutritional Biochemistry Core, Food and Nutrition Program, UNIRIO, Rio de Janeiro, RJ, Brazil; ^2^Laboratory of Proliferation and Cell Differentiation, Institute of Biomedical Sciences, Federal University of Rio de Janeiro, Rio de Janeiro, RJ, Brazil; ^3^Laboratory of Genomics, Molecular and Cellular Biology Program, UNIRIO, Rio de Janeiro, RJ, Brazil; ^4^Laboratory of Technology Natural Products, UEZO, Rio de Janeiro, RJ, Brazil

## Abstract

Breast cancer is one of the most prevalent cancers in the world and is also the leading cause of cancer death in women. The use of bioactive compounds of functional foods contributes to reduce the risk of chronic diseases, such as cancer and vascular disorders. In this study, we evaluated the antioxidant potential and the influence of pitaya extract (PE) on cell viability, colony formation, cell cycle, apoptosis, and expression of BRCA_1_, BRCA_2_, PRAB, and Er*α* in breast cancer cell lines (MCF-7 and MDA-MB-435). PE showed high antioxidant activity and high values of anthocyanins (74.65 ± 2.18). We observed a selective decrease in cell proliferation caused by PE in MCF-7 (ER^+^) cell line. Cell cycle analysis revealed that PE induced an increase in G_0_/G_1_ phase followed by a decrease in G_2_/M phase. Also, PE induced apoptosis in MCF-7 (ER^+^) cell line and suppressed BRCA_1_, BRCA_2_, PRAB, and Er*α* gene expression. Finally, we also demonstrate that no effect was observed with MDA-MB-435 cells (ER^−^) after PE treatment. Taken together, the present study suggests that pitaya may have a protective effect against breast cancer.

## 1. Introduction

Breast cancer is the most frequently diagnosed type of cancer around the world [1], and it is a complex disease caused by progressive genetic mutations, associated with other factors [2]. Various complications, including deaths from the disease associated with breast cancer, are due to metastasis. The rates of metastasis and mortality in breast cancer patients have decreased because of early diagnosis by mammographic screening and the implementation of adjuvant therapy. Currently, breast cancer control primarily involves surgical procedures and radiotherapy and is often supported by adjuvant chemotherapy or hormone therapies. This disease is highly resistant to chemotherapy, and there is still no effective cure for patients with advanced stages of the disease, especially in cases of hormone-independent cancer [3].

Several evidences, supported by epidemiological studies, indicate that prolonged exposure to sex hormones is one of the well-defined risk factors for breast cancer [4, 5]. Despite the fact that the majority of breast cancers are ER^+^, and hormonal intervention is used to prevent disease recurrence and/or progression, the mechanisms through which estrogen contributes to malignant transformation of mammary epithelium are poorly understood. ER^−^ tumors are associated with a worse short-term prognosis [6] and have weaker associations with reproductive risk factors [7] than ER^+^ tumors. Mutations in BRCA_1_ are associated with predisposition to ER^−^ breast tumors, whereas most known common susceptibility loci for breast cancer show stronger associations with ER^+^ than with ER^−^ tumors [8].

Carcinogenesis process results in the dysfunction of several regulatory features that keep the cells in check [9]. The balanced diet, with the diversified consumption of fruits and vegetables, exposes the body to several phenolic compounds. Over the last decade, these compounds have been widely studied and associated with benefits to human health. However, as there is a wide range of vegetables, species varieties, and differences in the compositions of these foods as well as the different localities of cultivation around the world, much research has yet to be done to elucidate the compounds present in these natural foods and their effective effects on the good health [10, 11].

Some reports support that the belief that components of food can affect the development of cancer in both beneficial and detrimental ways [12, 13]. Healthy lifestyle changes, including a better diet and regular exercise, can prevent up to 40% of breast cancers [14]. The role of fresh fruits and vegetables is to help prevent or lessen the action of free radicals [15].

The pitaya is also known as the “dragon fruit,” since it has a bright red peel with overlapping green fins that cover the fruit, a fact that has gained popularity in different countries of the world [16]. *Hylocereus polyrhizus*, which has red-skinned fruits with red meat, *Hylocereus undatus* (red pitaya), which has red-skinned fruits with white flesh, and *Hylocereus megalanthus* (yellow pitaya), which has yellow skin, are the most commercialized and consumed [17]. Red dragon fruit (*Hylocereus polyrhizus*) or sometimes called red pitaya has been comprehensively researched for its bioactive compounds.

Many compounds present in pitaya are responsible for many pharmacological activities such as antitumor, antioxidant, and anti-inflammatory actions. Bioactive compounds have been reported to modify specific carcinogenic processes, including cancer metabolism, hormonal balance, transcription factors, cell cycle control, apoptosis, inflammation, angiogenesis, and metastasis [18]. Potential mechanisms for cancer prevention of bioactive compounds in fruits include prevention of DNA adduct formation, enhanced carcinogen elimination, decrease inflammatory processes, and a direct cytotoxic effect on tumor cells [19, 20].

Recent reports have indicated that pitaya extract may have a role in the prevention and treatment of breast cancer [3, 21]. However, further studies on their role in the chemoprevention of breast cancer are warranted. In this context, the aim of the study was to evaluate the antiproliferative and proapoptotic effects of pitaya extract in MCF7 (ER^+^) and MDA-MB-435 (ER^−^) cell lines.

## 2. Methods

### 2.1. Sample and Extraction

The red pitaya (*Hylocereus polyrhizus*) were obtained from Petropólis (Rio de Janeiro State, Brazil). Hydroalcoholic extract was obtained from the pulp of the fruits. Fruits were washed in tap water, and the pulp was separated from the skins and seeds. Approximately 50 g of pulp of pitaya was extracted with 50 mL of ethanol and 50 mL of distilled water and then shaken for 2 h. After the pulp maceration period, the hydrohalic extract of pitaya was filtered on Whatman number 1 filter paper and the residual ethanol was evaporated under low pressure at 55°C. The extracts were then lyophilized and frozen at −20°C for use in the other experiments. Usually, 50 g of pulp yields 3 g of lyophilized extract.

### 2.2. Anthocyanin

Anthocyanins were extracted according to the method described by Abdel-Aal et al. [22] with slight modifications. Initially, 1 g of pitaya was extracted twice by mixing with 30 mL of methanol acidified with 1.0 N HCl (85 : 15, *v*/*v*) and shaking on a shaker at 4°C for 24 hr. The crude extracts were filtered with Whatman number 1 paper. The filtrate absorbance readings were taken at 535 nm, in Turner Model 340 spectrophotometer. To determine the anthocyanin values, we considered the dilution coefficients and the extinction coefficient of cyaniding 3-galactoside (98.2).

### 2.3. Antioxidant Activity Analyses

#### 2.3.1. Oxygen-Radical Absorbance Capacity Assay (ORAC)

The ORAC procedure used an automated plate reader (SpectraMax i3x, Molecular Devices, USA) with 96-well plates [23, 24]. Experiments were conducted in phosphate buffer pH 7.4 at 37°C. Peroxyl radical was generated using 2,2′-azobis (2-amidino-propane) dihydrochloride which was prepared fresh for each run. Fluorescein was used as the substrate. Fluorescence conditions were as follows: excitation at 485 nm and emission at 520 nm. The standard curve was linear between 0 and 50 mM Trolox. Results are expressed as *μ*mol TE/g.

#### 2.3.2. Ferric Reducing Ability (FRAP)

The extracts were measured for antioxidant activity by FRAP according to Rufino et al. [25]. Aliquots of 2.7 mL of TPTZ reagent (ferric 2,4,6-tripyridyl-s-triazine) were mixed with 0.5 mL of sample extract. After 30 min at 37°C temperature, the absorbance was read at 595 nm. The antioxidant capacity (FRAP) was expressed as Fe^3+^ equivalents (*μ*mol Fe^3+/^g dry basis).

#### 2.3.3. DPPH Assay

Aliquots of 0.5 mL of the extracts were mixed with 2.5 mL DPPH methanolic solution (0.06 mM) and allowed to react for 1 hour, in the dark. Measurements were performed at 515 nm applying a Turner 340 spectrophotometer. Analysis was performed in triplicates, and the decline in the DPPH radical absorbance concentration caused by the extracts was compared to a Trolox standard. The results were expressed as *μ*mol Trolox equivalents/g dry basis [19].

### 2.4. Cell Culture and Treatment Protocol

Cell lines were obtained from the Rio de Janeiro Cell Bank that certified their identity and quality (INMETRO—Rio de Janeiro, RJ, Brazil). Human breast adenocarcinoma cell lines (MCF-7 and MDA-MB-435) were plated in 25 cm^2^ tissue culture flasks (5.0 × 10^6^ cells/flask) and maintained routinely in the Dulbecco's modified Eagle's medium—high glucose (DMEM) supplemented with 10% fetal bovine serum (FBS) and 1% penicillin (PS), pH 7.4, under 5% CO2 atmosphere. Stock flasks were grown to 70% confluence and subcultured routinely. Medium renewal was done 3 times weekly. For each experiment, cells were seeded at 3.5 × 10^5^ cells/cm^2^ density in 6 and 2 × 10^4^ cells/cm^2^ densities in 96-well plates for cell cycle and cell proliferation analyses, respectively. After 24 h, medium was removed and cells were treated with increasing concentrations of PE (500 and 1000 *μ*g/mL) dissolved in DMEM. The controls, DMEM and DMEM+ 2% DMSO, were included on each plate. The cells were then incubated for 24 and 48 hours.

### 2.5. Cell Viability Assay

#### 2.5.1. MTT Assay

The status of cancer cell line viability was determined by the MTT (3-[4,5-dimethylthiazol-2-yl]-2,5-diphenyltetrazolium bromide; thiazolyl blue) assay (Sigma, New York, USA) wherein the substance is a pale yellow substrate that is reduced by living cells to yield a dark blue formazan product. This requires active mitochondria, and even recently, dead cells do not reduce significant amounts of MTT. Exponentially growing cells were adjusted to 2.0 × 10^4^/cm^2^ with DMEM, plated in 96-well plates (Corning, Tewksbury, MA) at 200 *μ*L/well, and incubated for 24 h according to the routine procedure. The cells were then incubated with PE (500 and 1000 *μ*g/mL) for 24 and 48 h. Each well was also incubated with MTT (10 *μ*L/well; 5 g/mL) for 4 h. At 85 *μ*L/well, the liquid was removed, and at 50 *μ*L/well, sodium dodecyl sulfate was added to dissolve the solid residue. Finally, the absorbance was measured using a microplate reader (POLARIS—CELER®) at 570 nm. The cell proliferation inhibition rate (CPIR) was calculated using the following formula: CPIR = (1 − average value of experimental group/average value of control group) × 100%.

#### 2.5.2. Test of Colony Formation (CFU)

Breast cancer cell lines were adjusted at a density of 10^3^ cells/per well in a 6-well plate in DMEM culture medium containing 10% FBS for 48 h. After this step, the cells were treated with PE at 500 and 1000 *μ*g/mL with medium replace every 5 days. After 18 days, colonies were fixed with 4% paraformaldehyde (Sigma, St. Louis, USA) in PBS containing 4% sucrose (Vetec, Rio de Janeiro, Brazil) for 20 min and then stained with 0.005% crystal violet (Vetec, Rio de Janeiro, Brazil) overnight at room temperature. For colonic analyses, they were washed five times with PBS for 5 min and 50 cells were counted using an Axiovert inverted microscope (Carl Zeiss, Oberkochen, Germany).

#### 2.5.3. Trypan Blue Exclusion Test of Cell Viability

Cells were grown to about 80% confluence in 6-well plates and treated for 24 h and 48 h with red PE at 500 *μ*g/mL and 1000 *μ*g/mL. Adherent and nonadherent cells were collected, and viability was assessed by mixing aliquots of cell suspensions with equal volumes of 0.4% trypan blue (GibcoBRL). Cells that accumulated the dye were considered dead.

### 2.6. Cell Cycle

Cells were rinsed briefly with calcium and magnesium-free phosphate-buffered saline and detached with trypsin at room temperature. After centrifugation, the cells were washed twice with phosphate-buffered saline and were resuspended in 500 *μ*L of ice-cold Vindelov solution [20] containing 0.1% Triton X-100, 0.1% citrate buffer and 0.1 mg/mL RNase, and 50 mg/mL propidium iodide (Sigma Chemical Co., St. Louis, MO). After 15 min of incubation, cell suspension was analyzed for DNA content by flow cytometry using a FACS Calibur flow cytometer (Becton Dickinson, Mountain View, CA). The relative proportions of cells with DNA content haploid subG_1_ (<2n), diploid G_0_/G_1_ (2n), S phase (>2n but <4n), and G_2_/M phase (4n) were acquired and analyzed using CellQuest and WinMDI 2.9, respectively. The percentage of cell population at a particular phase was estimated with FlowJo software following the acquisition of 30,000 events. Cell dissociation procedure does not affect fluorescence under the experimental conditions that were used in this study or in any others of which we are aware. Nuclei of viable cells were gated according FL-2 W **×** FL2-A relation.

### 2.7. Apoptosis Assay

Cells were resuspended in 400 *μ*L of binding buffer containing 5 *μ*L of annexin V FITC and 5 *μ*L propidium iodide (Apoptosis Detection Kit II, BDBiosciences) for 15 min at room temperature. Annexin V binding was evaluated by flow cytometry (FACScalibur, BD Biosciences), and after acquisition of 30,000 events, the data were analyzed in CellQuest and FlowJo software.

### 2.8. Gene Expression Analysis

Total RNA was extracted from the studied cells using Trizol® Reagent (Invitrogen) according to the manufacturer's instructions. RNA yield and quality were determined by a spectrophotometer Nano-Drop ND-1000 V3.2 (Nanodrop Technologies, Wilmington, DE). Equal amounts (500 ng) of RNA from cells were reverse transcribed with cDNA synthesis kit “Superscript II First-Strand Synthesis System for RT-PCR” (Invitrogen) and Oligo (dT) primer (Invitrogen). The cDNA was used as a template for subsequent real-time polymerase chain reaction (RT-PCR). Quantitative RT-PCR was done in a StepOnePlus™ Real-Time PCR System (Life Technologies) using SYBR Green (Applied Biosystems, Grand Island, NY) following the manufacturer's instructions and using primers as shown in Table 1. The expression levels of ERBB2, GSTM1, BRCA_1_, BRCA_2_, PRAB (progesterone receptor isoform A and B), ER*α* (estrogen receptor *α*), and GPR30 (a G protein-coupled receptor for estrogen) mRNA were all normalized with *β*-actin and GADPH (glyceraldehyde-3-phosphate dehydrogenase) expression level. For the evaluation of the quality of RT-PCR products, analyses of the melt curve were performed after each assay. The expression is relative to the measure using the ΔΔCT technique with *β*-actin and GADPH genes as the reference genes.

### 2.9. Statistical Analysis

The results presented are the mean and the corresponding standard deviation of three independent experiments performed in triplicate (*n* = 9). Data were analyzed using GraphPad Prism statistical software (version 5.04, GraphPad software, San Diego, CA). The univariate analysis of variance (ANOVA) with the Tukey posttest at a 95% confidence level was used to test cell viability, cell cycle, and apoptosis rate.

## 3. Results

### 3.1. Bioactive Properties of Red Pitaya

Natural and synthetic antioxidants are widely used in modern medicine. In the comparison of the antioxidant assays, an important bioactive potential in pitaya (10 mg/mL) was identified in ORAC values (1079.70 ± 75.20 *μ*M Trolox/g), FRAP assay (2519.36 ± 53.99 *μ*mol sulfate ferrous/g), and DPPH reduction (83.99 ± 0.30%) (Table 2). There is a need for screening studies in order to identify the mode of action of different antioxidant compounds (enzymatic and nonenzymatic in addition, comparing between synthetic and natural antioxidant compounds) by different assays [26].

Pitaya contained significant levels of total anthocyanins (Table 2). The pulp showed a significantly higher anthocyanin content (19.14 ± 0.52 mg/g) in comparison with peel (8.36 ± 2.70 mg/g).

### 3.2. Effect of Pitaya Extract (PE) on Cell Viability

#### 3.2.1. MTT Assay

**Figure 1:**
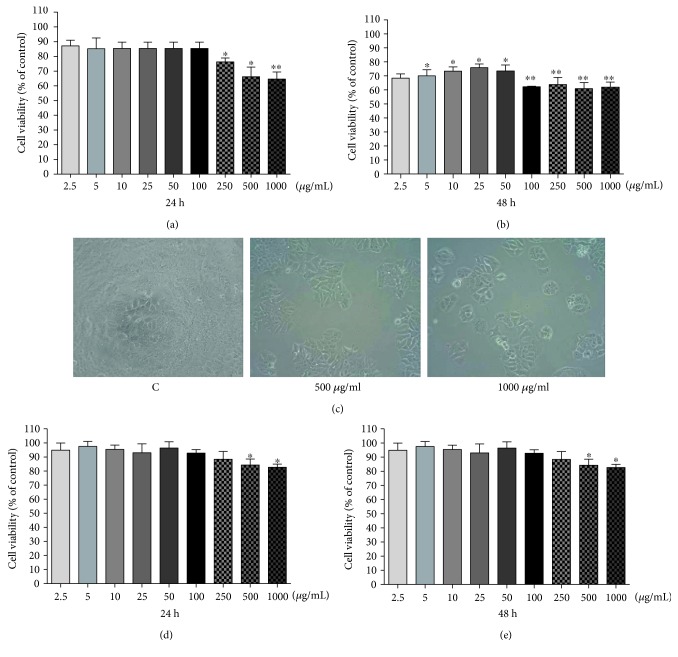
Effect of PE (2.5–1000 *μ*g/mL) on viability of MCF-7 (a, b) and MDA-MB-435 (d, e) cells at different time intervals after exposure using MTT assays. The experiment is expressed as mean ± standard error, and differences significant between treated cells with PE were compared using the Tukey test (^∗^*p* < 0.05; ^∗∗^*p* < 0.01). Phase contrast microscopy of MCF-7 cells (treated for 48 h with 500 and 1000 *μ*g/mL of PE) was observed on 96-well culture plates (c).

The treatment with PE for 24 h decreased MCF-7 cell viability from the concentration of 250–1000 *μ*g/mL, showing a mean reduction around 25.15% (*p* < 0.05) (Figure 1(a)). After 48 h, PE induced a higher inhibition of cell viability from the concentration of 2.5 *μ*g/mL (by 29.33% compared with the control group, *p* < 0.05), and the maximum inhibition was obtained with 1000 *μ*g/mL (40.22%, *p* < 0.05) (Figure 1(b)). Our data showed an important cell growth inhibition on MCF-7 cell after PE treatment (500 *μ*g/mL and 1000 *μ*g/mL) (Figure 1(c)).

As shown in Figures 1(d) and 1(e), a slight decrease in MDA-MB-435 cell viability was observed only in high concentrations of PE (500 and 1000 *μ*g/mL) with maximum inhibition of 20% compared with control group after 48 h (*p* < 0.05).

#### 3.2.2. Test of Colony Formation (CFU)

**Figure 2:**
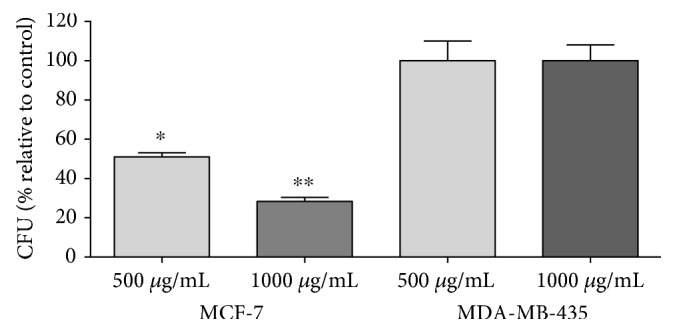
Formation of MCF-7 and MDA-MB-435 colonies. The number of MCF-7 and MDA-MB-435 colonies was determined after 18 days of culture in DMEM supplemented with 10% FCS containing PE at concentrations of 500 and 1000 *μ*g/mL. Data are presented as mean ± standard deviation of 3 independent experiments, each performed at least in duplicate. ∗indicates significant differences from the control group (^∗^*p* < 0.05; ^∗∗^*p* < 0.01).

The next step was to analyze the effect of PE on the clonogenic property of MCF-7 and MDA-MB-435 cells. According to the literature, cell groups with fewer than 50 cells were not considered as colonies [27]. Our data showed that the clonogenic ability of MCF-7 cells was inhibited in the presence of PE (500 and 1000 *μ*g/mL) (Figure 2). Maximum reduction of clonogenic ability was obtained when 1000 *μ*g/mL of PE (about 70%, ^∗∗^*p* < 0.001) was used (Figure 2). No effect in colony formation was observed in MDA-MB-435 cell line after PE incubation.

#### 3.2.3. Trypan Blue Exclusion

**Figure 3:**
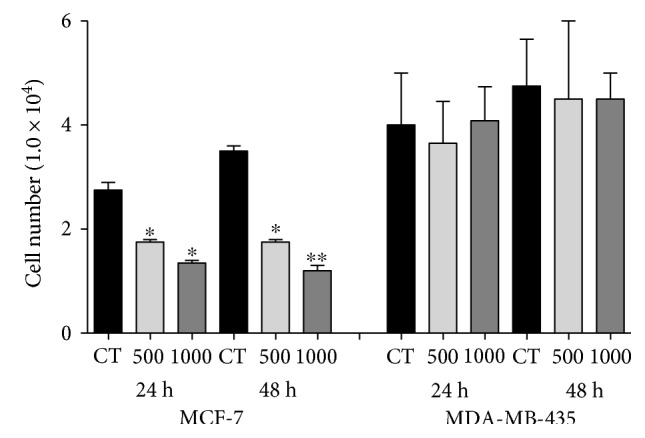
Effect of PE on cell proliferation of MCF-7 and MDA-MB-435 after 24 hours and 48 hours treatment using trypan blue exclusion. Data are presented as mean ± standard deviation of 3 independent experiments, each performed at least in duplicate. ∗ indicates significant differences from the control group (^∗^*p* < 0.05; ^∗∗^*p* < 0.01).

Another assay for cell proliferation was used to confirm the effect of PE in breast cancer cell lines. PE induced an inhibition of proliferation in MCF-7 cell line after 24 and 48 h from the concentration of 500 *μ*g/mL (by 50% compared with the control group, *p* < 0.05), and the maximum inhibition was obtained with 1000 *μ*g/mL (80%, *p* < 0.05). Corroborating with other methods used, when MDA-MB-435 cells were treated with PE for 24 h and 48 h, no changes in cell proliferation were detected when compared to untreated cells (Figure 3).

### 3.3. Effect of Pitaya Extract on Cell Cycle Progression

**Figure 4:**
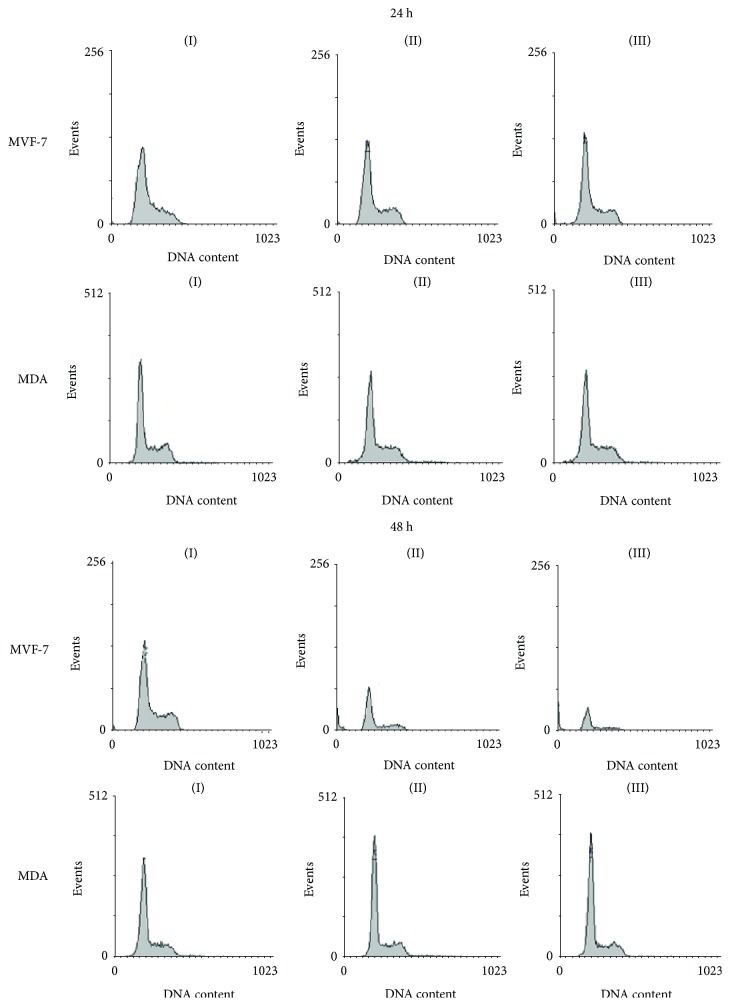
Effect of PE on cell cycle progression in MCF-7 and MDA-MB-435 cells after 24 and 48 h exposure. Data are presented as mean ± standard deviation of 3 independent experiments, with significant differences between the untreated cells (I) and treated with PE 500 (II) and 1000 (III) *μ*g/mL compared by the Tukey test.

We next questioned whether PE would have any effect on cell cycle arrest in breast cancer cell lines. After 24 h and 48 h of treatment, PE caused an increase in the percentage of cells in the G_0_/G_1_ phase, with a corresponding decrease in the G_2_/M phase, indicating a growth arrest of MCF-7 cells after that time (Figure 4 and Table 3). Corroborating with the data from cell proliferation, after 24 h and 48 h of treatment with PE, no changes in cell cycle profile of MDA-MB-435 cells were detected when compared to untreated cells (Figure 4 and Table 3).

### 3.4. Effect of Pitaya Extract on Apoptosis Assay

**Figure 5:**
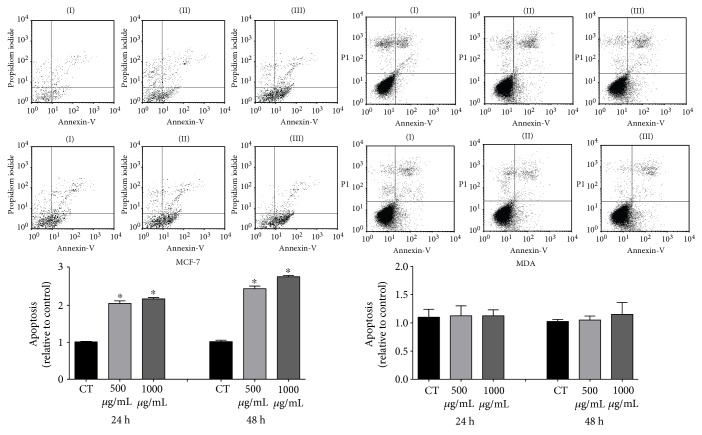
Detection of apoptotic MDA-MB-435 and MCF-7 cells by flow cytometry under PE stimulation at the concentrations of 500 and 1000 *μ*g/mL for 24 h and 48 h. Data are expressed as mean ± standard deviation relative to the control, of 3 independent experiments, each performed with at least 3 replicates. ∗ indicates significant differences from the control group (^∗^*p* = 0.05).

Flow cytometry analysis showed that treatment for 24 h and 48 h with PE at concentrations of 500 and 1000 *μ*g/mL did not induce apoptosis in MDA-MB-435 cells. However, when MCF-7 cells were treated under the same conditions for 24 and 48 h, an increase in the number of apoptotic cells was detected (Figure 5).

### 3.5. Gene Expression Profile

**Figure 6:**
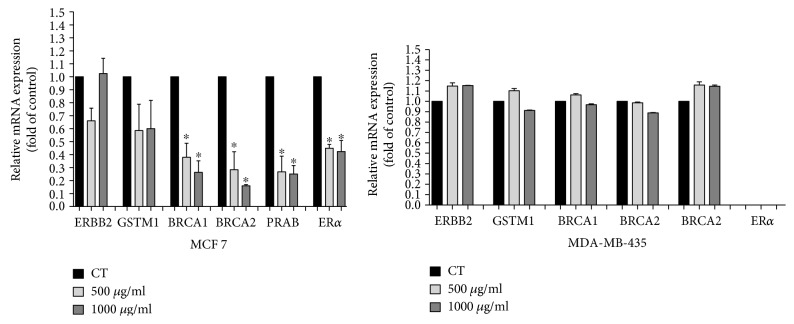
Profile of gene expression in MCF-7 and MDA-MB-435 cells. Quantitative analysis of real-time PCR in different genes associated with cancer progression, after 48 h incubation with PE. Data are presented as mean ± standard deviation of 3 independent experiments, each performed at least in triplicate. Differences significant between treated cells with PE (500 and 1000 *μ*g/mL) were compared using the Tukey test (^∗^*p* < 0.05).

The role of BRCA_1_, BRCA_2_, PRAB, and Er*α* genes as an oncogene responsible for the downregulation of the incidence of cancer progression is well established in a wide variety of tumors, including breast tumors. To study molecular mechanisms by which PE interferes in breast cancer progression, we investigated expression profile of several related genes (Figure 6). In MCF-7 cell line, PE treatment promoted a downregulation of BRCA_1_, BRCA_2_, PRAB, and Er*α* genes. Conversely, in MDA-MB-435 cells, no changes in gene expression profile cells were detected when compared to untreated cells (Figure 6).

## 4. Discussion

The present study provided several sets of information on the antioxidant activity of PE and their effects on the cell viability, cell cycle, and apoptosis of MCF-7 and MDA-MB-435 cells. Breast cancer is the most common cause of cancer in women and the large international variation in breast cancer rates, coupled with the rapidly increasing rates observed in secular trend studies. Although dietary factors have long been suspected to be implicated in breast cancer etiology, few convincing dietary risk factors have been identified [6]. Fruits and vegetables contain numerous constituents that may reduce breast cancer risk, including antioxidants and several vitamins which can prevent cancer [28].

The red pitaya features functional potential related to its high antioxidant activity [21]. Hylocereus species were responsible for the major antioxidant capacity [29], and some studies showed that the peels also contain more or less antioxidant properties due to their color. Thus, both the peels and the pulps could be beneficial especially in food and pharmaceutical industry [30]. The main mechanism of antioxidant action in foods is radical scavenging activity. Therefore, many methods had been developed in which the antioxidant activity was evaluated by the scavenging of synthetic radicals in polar organic solvents such as ethanol [17].

In previous studies evaluating extracts of other fruits by ORAC assay, it reported lower ORAC values than those found in this study. The antioxidant capacity of the hydroalcoholic concentrated extract of red grape pomace showed 22.94 *μ*M of Trolox/g for the ORAC assay. Already concentrate pitaya extract (PE) showed high antioxidant capacity with a reduction of up to 1000 *μ*mol Trolox/g^−1^ [31]. The US Department of Agriculture [32] published, as part of the National Programme for Food and Nutrient Analysis, a study containing data on the antioxidant capacity of concentrated fruit extracts, using the ORAC method. Among the tested fruits were the blackberry (88.57 *μ*M of Trolox/g), raspberries (37.98 *μ*M of Trolox/g), and a strawberry (32.26 *μ*M of Trolox/g).

FRAP is the only assay that directly measures antioxidants in a sample. The other assays are indirect because they measure the inhibition of reactive species (free radicals) generated in the reaction mixture, and these results depend strongly on the type of reactive species used. Mancini-Filho et al. [33] showed that those with average FRAP values higher than those found in the literature for other fruit extracts are also considered high potential antioxidants. The reducing potential of PE in this study was higher than the antioxidant capacity of some concentrated extracts of nontraditional Brazilian fruits such as camu-camu and uvaia jambolan. The fruits of camu-camu showed the highest antioxidant capacity, with a value of 2501.5 ± 74.5 *μ*mol sulfate ferrous/g. Acerola and the netting-black are also significant because the camu-camu showed the highest values, 1995.8 ± 47 and 28.4 ± 908.95 *μ*mol sulfate ferrous/g, respectively. The fruits of jambolan (172.8 ± 10.8 *μ*mol sulfate ferrous/g) and uvaia (407.5 ± 34.9 *μ*mol sulfate ferrous/g) showed lower values than those of pitaya.

Breast cancer cell lines MCF-7 and MDA-MB-435 are well known and widely used in studies on growth properties, regulatory mechanisms, and therapy of breast cancers. Our results showed for the first time that PE shows antitumorigenic effects on hormonal receptor-positive breast cancer MCF-7 cells. The epithelial cell line MCF-7 shows estrogen and progesterone receptors and low metastatic potential. Holliday and Speirs classified MCF-7 as cell line luminal with ER^+^, PR^+/−^, HER2^−^, and Ki67 low endocrine responsive and often chemotherapy responsive [34].

Recently, Wang et al. [35] demonstrated that differences between MCF-7 and MDA-MB-435 in 229 genes were mainly implicated in the biological functions related to cell adhesion and motion, antigen processing and presentation (via MHC class II), hormone response, extracellular structure organization, tissue remodeling, and cell proliferation regulation. A microarray analysis has indicated that the gene expression pattern of the human MDA-MB-435 [4] resembles that of human melanoma cell lines [5, 36]. This cell line has fusiform morphology and is considered luminal with low degree of invasion in Matrigel. The epithelial cell line MDA-MB-435 does not express hormone receptors and has a high metastatic potential and high tumorigenicity [37].

According to Ge et al. [38], MDA-MB-435 cell line is resistant to drugs in vitro breast cancer, due to the presence of high levels of *GSTP1* mRNA expression when compared to the levels expressed in MCF-7. Patients with breast cancer with the allele *GSTP1 105Val* are more likely to have a tumor with advanced histological grade, lymph node metastases, and negative estrogen receptor. The toxic damage to the genomic DNA in somatic cells not only induces carcinogenesis but also means that there is the development of tumors with more aggressive features, with poor differentiation, independent growth hormones, and metastatic potential. Probably, this is due to difference in the characteristic of aggressiveness between MCF-7 and MDA-MB-435 cell lines, since the MCF-7 cell line has hormone receptors and is more sensitive to the action of therapeutic drugs.

Pitaya has recently drawn much attention, not only because of their striking color and economic value as food products but also for their health properties [39]. For example, red pitaya was reported to offer many health benefits including chemoprevention of cancer, anti-inflammatory and antidiabetic effects, and a reduction in the mortality risk of cardiovascular disease [40], as well as antioxidative properties conferred by its betacyanin content [41]. Asmah et al. [42] reported that a red and white pitaya pulp are rich in polyphenols and a methanol extract showed promising antioxidant and antiproliferative capacity when used to treat cervix cancer cells (HeLa) and cytotoxic effect on human oral cancer cell metastases induced by B16-F10 melanoma.

Cell cycle deregulation is a fundamental aspect in cancer development. Deregulation of cell cycle has been linked with cancer initiation and progression [43]. Thus, cell cycle has emerged as one of the attractive therapeutic targets in the treatment of cancer [44].

Neoplastic cells contained in cell proliferation with a large proportion of cells in S phase and G_2_/M [45]. The efficiency of a bioactive compound in food cancer control can be judged by its ability to block the cell cycle phases G_0_/G_1_ and G_2_/M, reducing the proportion of cells in S phase [46]. PE promoted an increase in the percentage of cells in the G_0_/G_1_ phase, followed by reduction of cells in the G_2_/M phase, indicating an arrest in the growth and proliferation of MCF-7 cells after this period. One of the important and limiting aspects of the cell cycle is cell progression in the first phase (G_1_) of the S phase, which has its control affected in cancer [47].

There is an urgent need to develop innovative ways to treat breast cancer that has become resistant to apoptosis therapies. Apoptosis in clinical practice is a potential target for therapeutic use of programmed cell death or to understand the mechanisms of resistance to radiotherapy and chemotherapy. When cells become old or damaged, they die by apoptosis, necrosis, or a combination of the two and are replaced with new cells. On the other hand, cancer cells are immortal since they are resistant to apoptosis. Chemotherapy kills cancer cells through apoptosis and/or necrosis [48].

According Sreekanth et al. [49], pitaya extract compounds (betacyanin and anthocyanin) and pigments act on K562 cells that lead to human chronic myeloid leukemia altering the integrity of the mitochondrial membrane, leading to leakage of cytochrome c, caspase activation, and nuclear disintegration. These biochemical changes are reflected in structural changes typical of cells undergoing apoptosis (programmed cell death).

In this regard, the findings presented here coupled to the dragon fruit extract inhibited the viability and proliferation of human breast adenocarcinoma MCF-7, and it was found that these bioactive compounds present in the dragon fruit also interfere in the distribution phases of the cell cycle. However, we did not find studies of pitaya extract effects on tumoral breast cells in the literature.

Other components have already been well characterized in pitaya and, along with anthocyanins, have been described with substances potentially beneficial to human health. Esquivel et al. [29] found out that betalains containing both phenolic and nonphenolic structures were responsible for the major antioxidant capacity of purple Hylocereus juices evaluated, while nonbetalainic phenolic compounds contributed only to a minor extent. It was once thought that betalains were related to anthocyanins (i.e., a flavonoid derivative), the reddish pigments found in most plants [50].

Estrogen stimulates proliferation of various breast cancer cells via estrogen receptors (ER). Studies show that different compounds present in food matrix could bind to estrogen receptors and mediate estrogen responses [51, 52]. The majority of authors show that there is a positive association between the presence of hormone receptors and a more favorable prognosis. The presence of hormone receptors indicates a functional state closest of normal breast cell. In other words, these tumors are similar in morphology to the cells of origin and thus are less aggressive to the body. The estrogen receptor expression by tumor cells suggests that at least part of cell proliferation depends on stimulation by estrogen. Therefore, it is possible to stop cell growth by blocking hormone [53]. The activity of PE was evaluated in this study to identify potential signaling pathways by real-time PCR analysis; the observations indicate that the PE showed antitumor activity in MCF-7 cell line by probably suppressing ER*α*.

BRCA_1_ and BRCA_2_ are human genes that produce tumor suppressor proteins. These proteins help repair damaged DNA and, therefore, play a role in ensuring the stability of the cell's genetic material. Genetic susceptibility to breast cancer comprises inherited mutations of the BRCA_1_ and BRCA_2_ genes related to hereditary breast cancers. In addition, some studies reported that vegetable and fruit intakes were modifiers in developing breast cancer in BRCA mutation carriers [54].

It is known that BRCA-related tumorigenesis is mainly caused by increased genome instability and DNA damage, but it is unclear why patients who have a mutation in BRCA_1_ BRCA_2_ are at higher risk of developing estrogen-responsive cancer. Literature suggests that BRCA_1_ and estrogen and estrogen receptor signaling regulate cell proliferation and differentiation of breast cells, synergistically [55].

BRCA_1_ and BRCA_2_ were downregulated upon pitaya treatment, indicating that DNA damage and repair pathways were affected. Proteins (PRAB, BRCA_1_, and BRCA_2_) playing role in DNA damage response pathway were deregulated upon pitaya treatment [56]. Downregulation of PRAB, BRCA_1_, and BRCA_2_ imply that uncontrolled proliferation was to some extent normalized and DNA damage was accumulated leading to apoptosis. Our results on pitaya extract can be reconciled with more general findings in cancer biology that tumors activate DNA damage response pathways such as BRCA_1/2_ upon exposure to DNA-damaging agents [57]. It is worth speculating that pitaya may be even more cytotoxic, if combined with other DNA-damaging drugs such as doxorubicin and cisplatin.

Thomson and Thompson [58] support the emphasis of public messages for greater vegetable and selective fruit intake by extending a potential benefit for ER-negative breast cancer. On the other hand, tumors with positive hormone receptors have a more favorable prognosis and respond better to hormonal therapy. This is because the strategies of treating a malignant tumor sensitive to hormones involve, on the one hand, the reduction of estrogen produced normally by the body and, on the other, the inhibition of the links between receptors and hormones. The first group has use drugs which inhibit the synthesis of the hormone, such as those that reduce the activity of the aromatase enzyme responsible for the synthesis of estrogens in various tissues, such as adipose tissue. Another option, more drastic and in selected cases, would be the surgical removal of the ovaries, which produce estrogens in premenopausal women. In the second group are drugs that aim to disrupt and/or compete with estrogens in its binding to the receptor.

**Figure 7:**
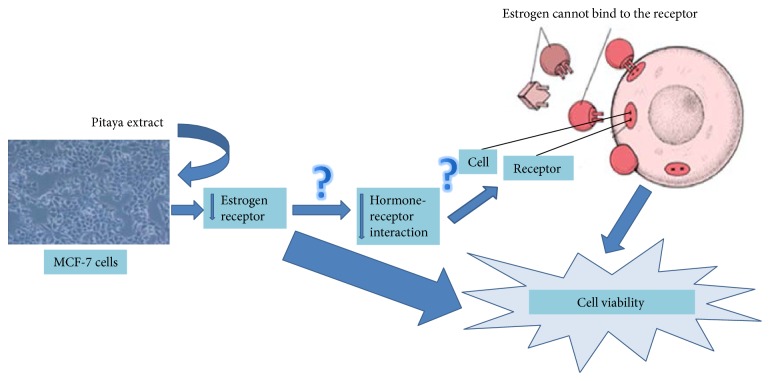
The proposed mechanism of action of PE in MCF-7 cells associated with decreased estrogen receptor expression.

Studies have shown that polymorphisms in the ER*α* gene (ER-alpha) are associated with diseases such as breast and prostate cancer, osteoporosis, Alzheimer's disease, and cardiovascular diseases [59]. The probable mechanisms of pitaya's proliferative action appear to be dependent on decreased ER*α* expression that can directly trigger mechanisms of inhibition of cell viability or perhaps decreasing hormone binding to the receptor and thereby inhibiting cell growth (Figure 7). More studies are needed to conclude that the effects of pitaya extract are truly ER-dependent.

## 5. Conclusion

We conclude that pitaya may act on selective ER-responsive breast cancer cells by targeting multiple tumorigenic pathways leading to cell cycle arrest and apoptosis and probably suppress the expression of estrogen and progesterone receptors. Our data indicate that pitaya possesses therapeutic potential against breast cancer. Further preclinical and clinical studies are warranted to clarify the therapeutic potential of pitaya in the prevention and adjuvant treatment of breast cancer.

## Figures and Tables

**Table 1:** Primer sequences for the reverse transcription-quantitative polymerase chain reaction.

Gene	Forward primer	Reverse Primer
ERBB2	CCGTGCCACCCTGAGTGT	AGCCTCCGGTCCAAAACAG
GSTM1	TCCCTCTTCACTCCCCCTAAA	GGGTAGCTGAGGCTTCAAAGG
BRCA_1_	CTGCTCAGGGCTATCCTCTCA	TGCTGGAGCTTTATCAGGTTATGT
BRCA_2_	CCACAGCCAGGCAGTCTGTAT	AGAACACGCAGAGGGAACTTG
PRB	CCTGAAGTTTCGGCCATACC	CAGGGCCGAGGGAAGAGT
PRAB	GGCTACGAAGTCAAACCCAGTT	CAATTGCCTTGATGAGCTCTCTAA
ER*α*	CTGTTTGCTCCTAACTTGCTCTTG	TCCACCATGCCCTCTACACA
GAPDH	ATGGAAATCCCATCACCATCTT	CGCCCCACTTGATTTTGG

**Table 2:** Bioactive potential of pitaya evaluated by different methods.

Pitaya	2.0 mg/mL	5.0 mg/mL	10.0 mg/mL	*R* ^2^
ORAC assay (*μ*M Trolox/g)	140.50 ± 1.90	560.00 ± 48.90	1079.70 ± 75.20	0.9943
FRAP assay (*μ*mol Fe_2_SO_4_/g)	909.20 ± 68.46	1698.64 ± 33.17	2519.36 ± 53.99	0.9621
DPPH assay (% reduction)	33.05 ± 0.32	73.01 ± 0.38	83.99 ± 0.30	0.8892

	Pulp	Peel	Total anthocyanins (fruit)	Total anthocyanins (pitaya extract)
Total anthocyanins (mg/g)	19.14 ± 0.52	8.36 ± 2.70	27.50 ± 1.61	74.65 ± 2.18

Results expressed in mean ± standard error.

**Table 3:** Effect of PE on cell cycle progression in MCF-7 and MDA-MB-435 cells after 24 h and 48 h exposure.

Cell line	Incubation time	Cell cycle phases	Control (CT)	500 *μ*g/mL	1000 *μ*g/mL
MCF-7	24 h	G_0_/G_1_	59.59 ± 0.16	63.47 ± 2.07^∗^	65.02 ± 0.23^∗^
		S	16.91 ± 1.20	14.03 ± 0.61	14.80 ± 2.36
		G_2_/M	19.28 ± 1.87	19.77 ± 1.42	17.48 ± 3.22
	48 h	G_0_/G_1_	58.49 ± 0.45	65.40 ± 1.10^∗^	69.61 ± 3.90^∗^
		S	16.45 ± 0.55	10.63 ± 0.25	13.64 ± 1.71
		G_2_/M	22.28 ± 0.93	20.19 ± 0.04^∗^	15.66 ± 3.72^∗∗^

MDA-435	24 h	G_0_/G_1_	62.30 ± 1.12	61.99 ± 1.99	61.60 ± 0.64
		S	14.84 ± 0.43	14.93 ± 0.24	14.87 ± 0.51
		G_2_/M	19.90 ± 1.29	19.83 ± 2.04	18.88 ± 0.68
	48 h	G_0_/G_1_	69.64 ± 1.18	70.64 ± 0.80	70.01 ± 1.85
		S	11.88 ± 0.89	11.30 ± 0.62	11.50 ± 0.53
		G_2_/M	15.17 ± 1.03	15.52 ± 0.52	15.76 ± 0.83

The cell cycle phases and quantitative results are illustrated in accordance with the exposure time and PE concentration. The experiment is expressed as mean ± error standard. ∗ indicates significant differences from the control group (^∗^*p* < 0.05; ^∗∗^*p* < 0.01).
